# Using a mark-recapture model to estimate beaching probability of seabirds killed in nearshore waters during the Deepwater Horizon oil spill

**DOI:** 10.1007/s10661-019-7919-9

**Published:** 2020-03-17

**Authors:** Gina K. Himes Boor, R. Glenn Ford

**Affiliations:** 1 Ecology Department, Montana State University, Bozeman, MT 59715 USA; 2 R.G. Ford Consulting Company, 2735 N.E. Weidler Street, Portland, OR 97232 USA

**Keywords:** Deepwater Horizon oil spill, Mark-recapture Brownie model, Seabird, Impact assessment, Gulf of Mexico, Carcass sinking rate

## Abstract

During a marine oil spill, injured birds often die on the water, some eventually washing ashore, but others becoming waterlogged and sinking or being scavenged before reaching the shoreline. Birds that disappear before they can be deposited on the shoreline are difficult to enumerate, but they commonly represent a large fraction of total oil spill–related mortality. As part of the process of quantifying the overall impact to seabirds resulting from the Deepwater Horizon oil spill, it was necessary to estimate the number of dead birds lost at sea. We conducted a study to estimate the beaching probability of birds that died at sea in the Gulf of Mexico in the areas most heavily used by seabirds and impacted by the spill. Using a mark-recapture analysis to derive the beaching probability from our field study data, we estimated that dead birds afloat at sea had about a 0.1414 probability of beaching in areas searched during the Deepwater Horizon oil spill. Sensitivity analyses of our model and requisite assumptions suggested that if our assumptions were violated, the “true” beaching probability could be anywhere between 0.11 and 0.16. These estimates are much lower than beaching probabilities estimated for seabirds killed during the Exxon Valdez oil spill in the waters of Alaska, for example, likely reflect higher rates of decomposition and scavenging in the warmer waters of the Gulf of Mexico. Our estimate suggests that bird carcasses that washed onshore during the Deepwater Horizon oil spill represented only 14% of those killed at sea during the spill.

**Electronic supplementary material:**

The online version of this article (10.1007/s10661-019-7919-9) contains supplementary material, which is available to authorized users.

## Introduction

Seabirds and waterbirds spend much of their time sitting on the water, flying over water, or resting along tidally inundated shorelines and sandbars. During an oil spill, injured birds often die in situations where they float for some period of time before beaching or succumbing to other processes (e.g., sinking, scavenging). Birds that disappear before they can be deposited on the shoreline are obviously difficult to enumerate, but other studies have shown they commonly represent a large fraction of total oil spill–related mortality (Ford et al. 1996; Helm et al. 2015). The importance of at-sea loss during an oil spill and subsequent Natural Resource Damage Assessments (NRDA) was recognized as far back as the Apex Houston spill in 1986, but at that time no data were available to determine how long carcasses floated before disappearing. For the Apex Houston, researchers assumed that the rate at which birds succumb to sinking was constant (e.g., if all birds died at the same time, the number of birds lost to sinking would follow an exponential decay function) starting as soon as the bird died (Carter et al. 2003).

Subsequent studies showed that carcasses placed in water-filled tanks floated for unrealistically long lengths of time, reaching levels of decomposition that were not observed in the field (Ford et al. 1991; Wiese 2003). However, carcasses that were tethered to anchors placed in open water tended to disappear rapidly as the decomposing flesh was stripped off by the pull of currents, leaving only articulated skeletons attached to their tethers (Ford et al. 1991). More realistic results were obtained by tethering carcasses to small floating buoys equipped with long-range VHF transmitters and self-righting antennae (Ford et al. 1996). These buoys were constructed with less than a gram of buoyancy, so that as decomposing carcasses became neutrally and then negatively buoyant, the buoys and antennae would follow suit and the VHF signal would attenuate and then disappear. Signals would also cease if carcasses were consumed by larger scavengers.

This general technique was utilized for assessing bird loss at-sea for oil spills in California, Oregon, and Alaska. Results indicated that carcasses floated for one to 3 weeks before beginning to sink. Thereafter, carcasses disappeared at an accelerating rate, rarely persisting much beyond 3 weeks. There was no evidence that carcasses were consumed by larger scavengers, but that may have occurred in some cases. During the response to the Deepwater Horizon spill, it was recognized that the fates of bird carcasses might differ greatly between the Gulf’s warm subtropical waters and the cold Pacific waters of earlier studies.

The present study was specifically designed to estimate the daily at-sea disappearance rate of bird carcasses (and the correlate beaching probability) during the Deepwater Horizon (DWH) oil spill, and to compare these rates with the earlier studies in the Pacific. To do so, we conducted a field study in which we tracked seabird carcasses in the Gulf of Mexico using telemetry, then used a mark-recapture model to analyze the field study data in the context of the conditions present during the DWH spill.

## Methods

### Carcass drift study data

**Fig. 1:**
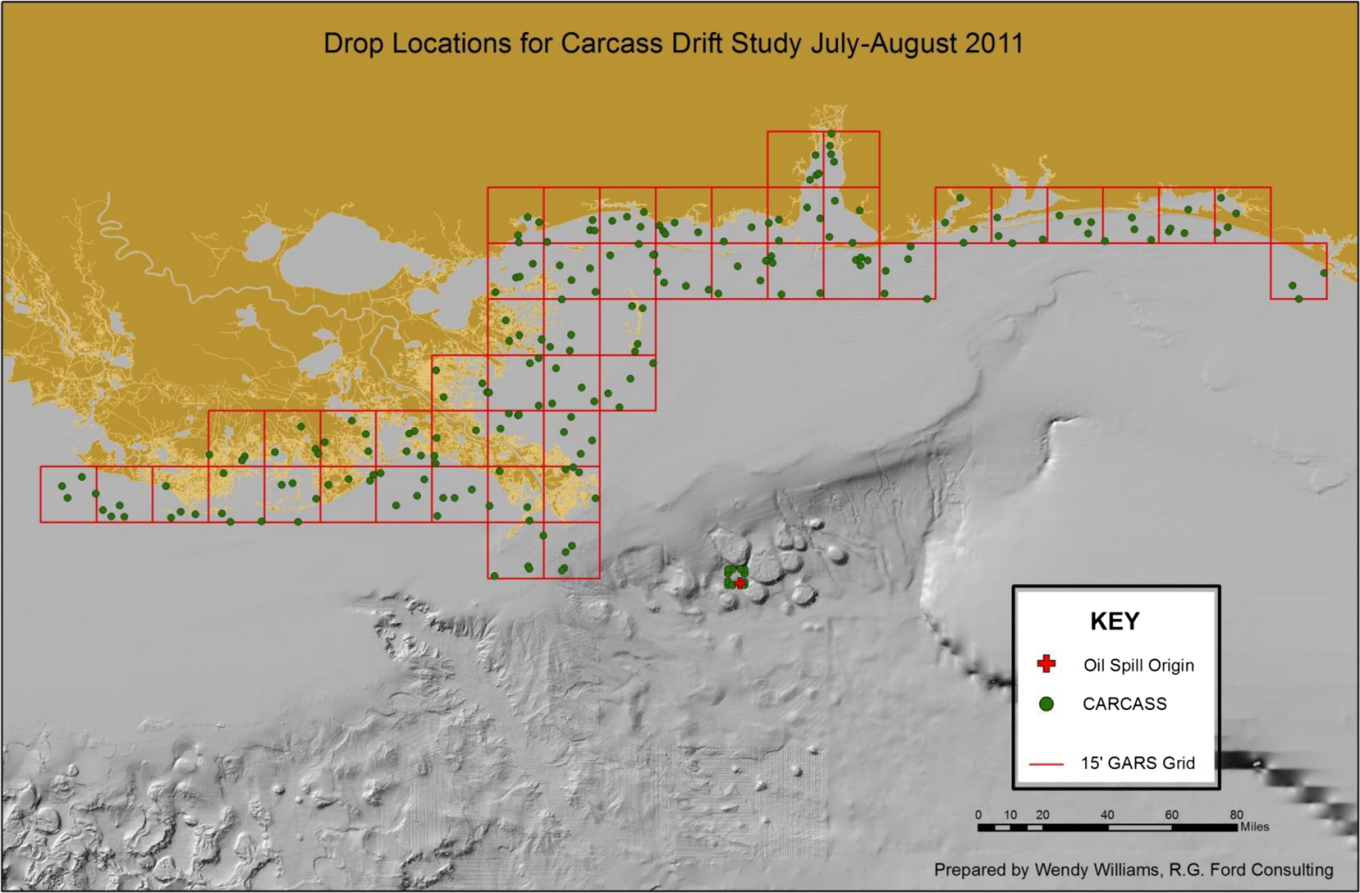
Map of study area and carcass assembly release locations

To determine the daily disappearance rate of bird carcasses during the DWH oil spill, we conducted a carcass drift study in the Gulf of Mexico in the summer of 2011, 1 year after the DWH spill. Below, we provide a brief overview of the field study from which we derived the data for the present analysis, but for a full description (including additional maps), see Ford et al. (2014b) and Martin et al. (2020). As part of the study, 200 carcasses attached to floats with radio transmitters (i.e., barges) were released in nearshore waters and tracked using radio telemetry. Carcasses were released in randomized locations within 50 of the 15’ GARS (Global Area Reference System; http://earth-info.nga.mil/GandG/coordsys/grids/gars.html) grid cells in the study area on 13 dates between July 15 and August 6, 2011 (see Ford et al. 2014a, b; Martin et al. 2020), so that four carcass assemblies were released within each GARS cell (Fig. 1). The 50 GARS cells used for these releases were those cells between Atchafalaya and Apalachicola that had the highest bird densities during the DWH spill as assessed during the NRDA aerial bird surveys conducted between May and September 2010 (Ford et al. 2014a, b).

Carcass assemblies (bird and transmitter barge) were relocated via telemetry, during the 35 days of the study via aircraft, boat, and ground crews to determine if and when a carcass arrived on shore within the study area. During the first weeks of the study, we used the NOAA spill model GNOME (Beegle-Krause 2001) combined with predicted wind and current fields for 2011. Assistance with model setup and access to real-time wind and current predictions was provided by members of the Hazardous Materials Branch (Watabayashi, pers. comm). This model was used in an attempt to predict where carcasses would beach, and thus, where our aerial overflights and boat crews should focus their efforts to locate carcass assemblies each day. After several weeks, however, we found that carcasses were as likely to move opposite the direction predicted by our spill model as with it. Our lack of success in predicting carcass movement implies that there was a high degree of variability in nearshore winds and currents that our modeling could not resolve. As a result, we abandoned the real-time predictive modeling and opted to search as much of the study area as possible each day with aircraft and boat. Using information from these air- and water-based searches, ground crews were directed to specific shoreline areas where telemetry signals were detected to search for potentially beached carcasses. Five of the 200 carcasses were picked up by citizens prior to ground crews reaching them, and their location and time of arrival onshore could not be determined. We therefore excluded these five carcasses from the analysis, leaving a total of 195 carcasses used in the analysis.

**Fig. 2:**
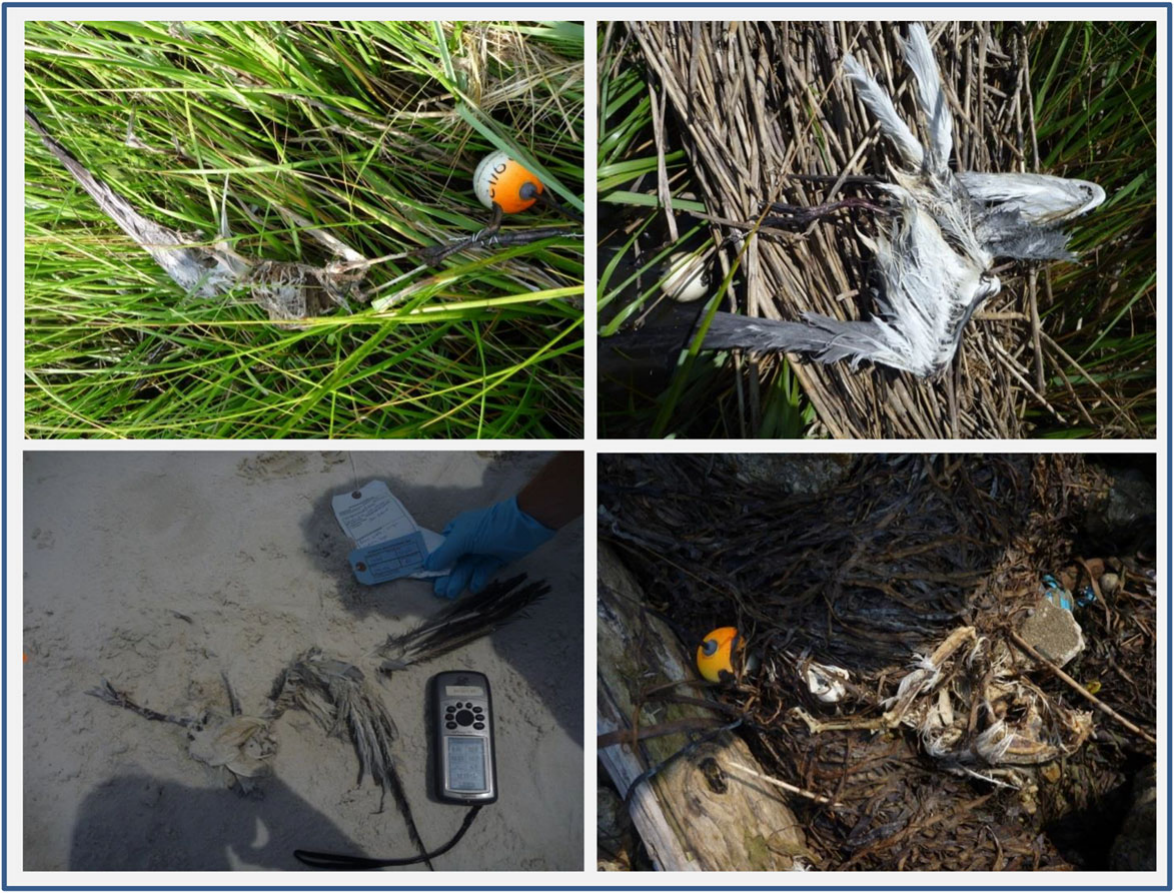
Examples of the state of carcasses found by ground crews during the carcass drift study that were considered to be recognizable as birds. Some were nearly intact carcasses, others were closer to piles of feathers and bones. A total of 35 carcasses came ashore in this state

The study area included all shoreline areas within the spill-affected GARS grid cells that were searched during the DWH spill response. Of the 195 carcass assemblies used in the analysis, 27 (13.8%) came ashore on land or in emergent vegetation (i.e., they “beached”) within the study area and were found by ground crews in a condition that resembled a bird, which we define as any carcass with more than just feet or legs (see Fig. 2). The remaining 168 carcasses were categorized as “permanently lost”. The lost fate could involve sinking, washing out to sea, being eaten, being buried, or beaching outside the study area. By tracking the carcasses with telemetry, in some of the 168 cases, we know which of these specific lost fates the carcass succumbed to; in other cases, we do not. Following is an enumeration of the specific fates of these carcasses and a brief discussion of why they were considered “lost” for the purposes of our analysis.

**Fig. 3:**
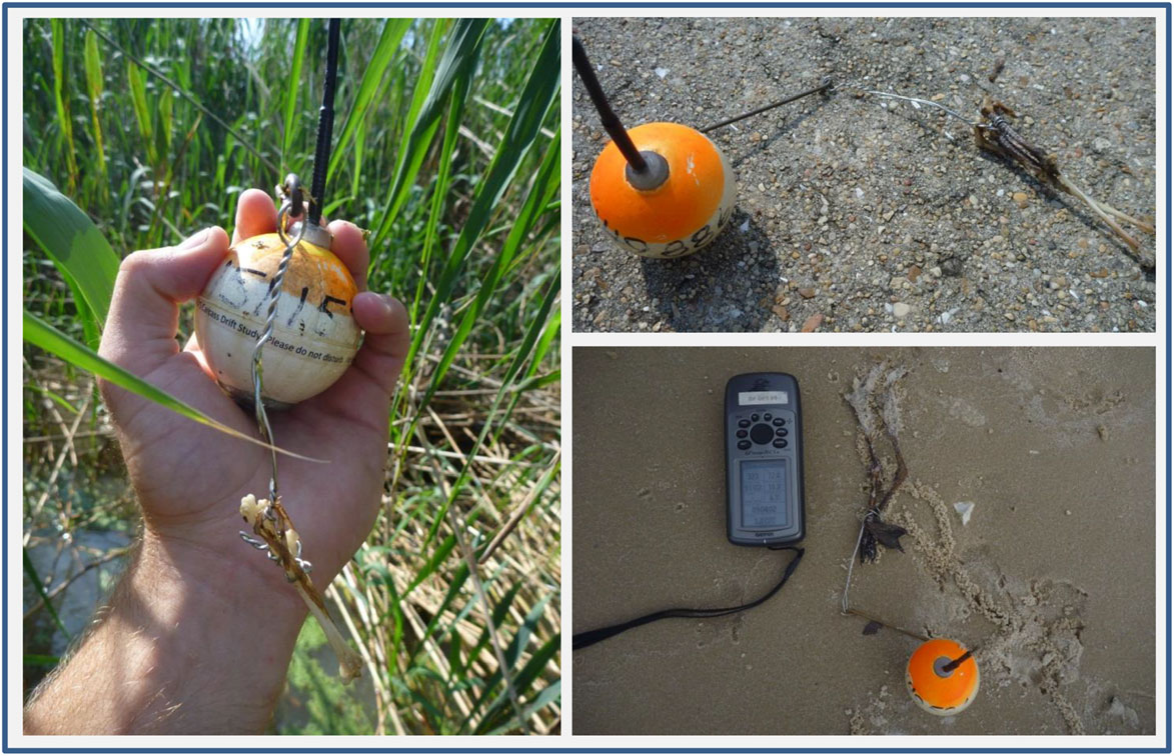
Examples of barges found by ground crews during the carcass drift study that were attached to foot or leg bones only. Such assemblages were categorized as “lost” for our analysis. There were 24 such barges found by ground crews during the study

Of the 168 lost carcass assemblies, 20 came ashore outside the study area. Since the purpose of the analysis was to determine the rate at which spill-affected carcasses beached within areas that spill-response personnel could collect them, drift study carcasses that landed onshore outside the study area were not counted as beached for the purposes of our study. Another 24 barges were found onshore by ground crews within the study area but were attached solely to foot or leg bones without any other remnant of the bird (i.e., did not “resemble a bird”; see Fig. 3). We assumed for this analysis that these 24 “foot-only” carcasses had been scavenged prior to coming ashore and that the barge attachment to the leg prevented the feet from being consumed with the rest of the carcass, or that the barge prevented the unconsumed feet from sinking, and therefore only arrived onshore because of the attached barge (see “Discussion” for more on this assumption). Another 15 barges were collected onshore with no remnant of the carcass to which they were originally attached. We assume these carcasses were completely scavenged prior to landing onshore. Of the remaining 109 barges, 18 were never detected by air, land, or water in the study area after their initial drop (assumed washed out to sea), 79 were detected via telemetry signal one or more times by air or boat but never made it to shore, 7 were located by boat- or land-based crews under water or deep sand, and 5 were detected via telemetry signal by search crews on or near shore but could not be located or retrieved. Barges that could not be located or retrieved were likely deeply buried, under water, or deep in rip-rap. Sunk or buried carcasses would not be findable under normal spill-response search conditions and were therefore considered permanently lost for our analysis.

Note that the present study does not deal with the “findability” of carcasses, e.g. whether or not a spill responder would have found and enumerated a carcass. The overall likelihood of a carcass being enumerated is the product of the probability that it would be beached where it could be found, and the probability that it would actually be found by searchers once there. Here, we attempt only to estimate the likelihood that a carcass would have beached where it could have been found; the findability issue is dealt with elsewhere (Martin et al. 2020; Varela et al. 2015).

### Model overview

A carcass afloat at sea has one of three possible fates on any given day: (1) it remains afloat at sea, (2) it beaches within the study area, or (3) it disappears permanently from the study area (i.e., is lost). Both fates 2 and 3 (beaching or being lost) are considered terminal fates. Once a carcass succumbs to fate 3 (is lost), it is no longer available to wash ashore. Similarly, for fate 2 (beaching), once a carcass beaches, we can record it as having made it to shore in the study area, which, for the purposes of this study is its final fate. In an actual spill situation, beached carcasses might or might not persist to be found by searchers, but those post-beaching processes (i.e., scavenging, tidal rewash, searcher efficiency) are not the focus of this study (but see “Discussion” for more on this).

To estimate the probability of each of the three daily carcass fates from our data, we used a mark-recapture approach called the Brownie band-recovery model (Brownie et al. 1985) implemented in Program MARK (White and Burnham 1999). This model uses the proportion of carcasses that were never found, along with the proportions that were found onshore on each day of the study, to estimate the daily probability of beaching, staying afloat at sea, or permanently being lost.

The Brownie model was originally designed to estimate annual survival probabilities using data from banded birds whose bands were returned to researchers when the birds were killed during hunting season. The data needed for the typical Brownie analysis are the number of bands deployed per year and the number of bands recovered from hunters in each subsequent year. From these data, the model is used to estimate the annual probability of surviving and the annual probability of recovery (i.e., being killed and reported as such).

**Fig. 4:**
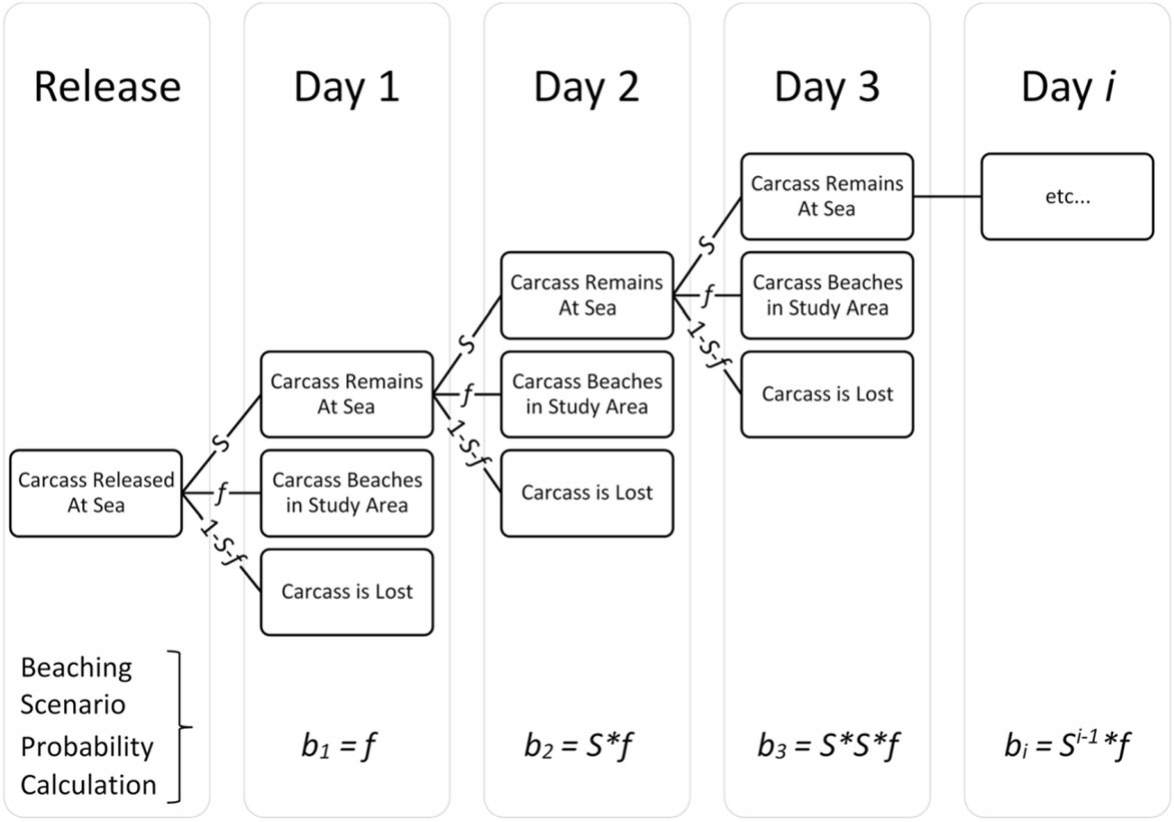
Schematic representation of each possible daily fate for a carcass floating at sea and the probabilities of each. If a carcass “survives” at sea (with probability *S*), it faces the same possible fates the following day—a process that continues until the carcass either beaches (*f*) or is permanently lost (1−*S*−*f*). The equations at the bottom represent the calculation of the probability of each beaching scenario (*b*_*i*_): beaching on day 1 (*b*_1_ *= f*), “surviving” at sea for one day then beaching on day 2 (*b*_2_ *= f × S*), “surviving” at sea for 2 days and beaching on day 3 (*b*_3_ *= f× S× S*)

In our analysis, the estimated survival rate (*S*) represents the probability of a carcass remaining afloat at sea (i.e., “surviving” at sea), and the recovery rate (*f*) represents the probability of a carcass beaching (Fig. 4). The data used for the model were the number of carcasses released (*n* = 195), the date of those releases, and the date of arrival onshore for each of the beached carcasses. The study lasted a total of 35 days. The time step in our analysis is equal to 1 day, and therefore, *S* represents the probability of a carcass surviving at sea for 1 day, and *f* represents the probability of a carcass beaching within the study area sometime over the course of 1 day. These two fates are mutually exclusive but not exhaustive outcomes. The third possibility on a given day is to disappear permanently, i.e., to sink, be buried, be eaten, or land onshore outside the study area. Together, these three daily fates represent all possible daily outcomes, so the daily probability of permanently disappearing can be calculated by subtracting the other two probabilities from 1, that is, the daily probability of being permanently lost (*ω*) is equal to 1−*S−f*.

Once the daily probability of each fate is estimated using the Brownie model, we calculate the overall probability of a carcass ever beaching within the study area (*β*). This requires the calculation of *b*_*i*_ for each day *i*, which is the probability that a carcass will survive at sea *i−*1 number of days and then beach on day *i*. For example, consider the probability that a carcass would be beached on day 3 (*b*_3_). The carcass would have to remain afloat at sea for the first day after being released (i.e. probability *S*), continue to float on the second day (again with probability *S*), and then beach on the third day (with probability *f*). The probability of this exact scenario (i.e., a carcass beaching 3 days after dying at sea) is calculated by multiplying the individual daily probabilities together, *b*_*3*_ *= S × S × f* (Fig. 4).

After calculating the probability of a carcass surviving and coming ashore on each day (i.e., day 1, day 2, and day 3), we can calculate the probability that any of those scenarios happened (i.e. the overall beaching probability of a carcass, *β*) by summing the probabilities of those individual scenarios. In other words, we estimate the probability that a bird that died at sea would come ashore on day 1 or day 2 or day 3 or … etc., which is equivalent to calculating the overall probability that a bird would come ashore at all after dying at sea (*β*). Although the carcass drift study only lasted 35 days, our model structure and parameter estimates allow us to calculate the probability of a carcass beaching after any number of days being out at sea.

### Candidate models

If daily survival or beaching does not depend on the day or the age of the carcass, then the model has only two parameters to estimate, *S*(*.*) and *f*(*.*), as described above. If, however, survival or beaching probabilities differ depending on date (*S*(*t*) or *f*(*t*)) or the age of the carcass (i.e., how long it had been afloat; *S*(age) or *f*(age)), the number of parameters in the model increases depending on the number of time or age classes specified. For example, if at-sea conditions were rougher on some days and those conditions affected carcass survival at sea, then a model with time-dependent (i.e., date-dependent) survival (*S*(t)) might be most appropriate. Or if, for example, a carcass is more likely to sink (and therefore not survive) the longer it remains at sea, then a model that incorporates carcass age (*S*(age)) might be most suitable.

We tested a total of 14 candidate models that included a variety of structures, from a 2-parameter model with constant daily survival and beaching probabilities across date and age of carcass (e.g., Model 1: *S*(.) *f*(.)), to fully time-dependent and age-dependent survival and beaching models (Table 1). The fully time-dependent models included individual survival and/or beaching probabilities for each date of the study (e.g., Model 14: *S*(t) *f*(t)). Because such a structure allows the model to account for date-specific factors such as strong wind or circulation patterns that might impact beaching or survival on any given day, and because our experience using wind and circulation patterns to predict real-time beaching vicinities during the field portion of the study was unsuccessful, we did not test any models with parameters specific to wind or circulation patterns.

**Table 1:** List of candidate models considered. Models are listed in the order from most- to least-supported based on their Akaike’s information criterion for small sample sizes (AIC_c_). The models are described by their estimated parameters: daily “survival” at sea (*S*; i.e., the daily probability of remaining afloat at sea) and the daily probability of beaching (*f*). *S*(.) and *f*(.) represent models with a single time- and age-invariant estimate for each parameter. *S*(*t*) and *f*(*t*) represent models with time-varying parameters. Models that include different survival and/or beaching probabilities for different aged carcasses include the word “age” (e.g., *S*(age) and *f*(age)) followed by the number of age-dependent parameters if the model is not fully age-dependent. Note that for models with a large number of parameters (e.g., those with full time or age dependence) not all parameters are statistically identifiable, meaning that some combinations of survival and beaching probabilities are estimable together (e.g., day 18 survival and day 18 beaching) but cannot be estimated individually. As a result, the number of parameters listed in the “No. parameters” column may be smaller than the total number of parameters that would be expected if all parameters were individually identifiable

Model no.	Model	AIC_c_	Delta AIC_c_	AIC_c_ Weights	Model likelihood	No. parameters	Deviance	− 2log(L)
1	*S*(.) *f*(.)	300.5363	0	0.25409	1	2	112.5035	296.4738
2	*S*(.) *f*(age-4 w/1st “age” = 2 days)	300.9173	0.381	0.21001	0.8265	5	106.6296	290.5998
3	*S*(.) *f*(age-2)	302.4312	1.8949	0.09852	0.3877	3	112.3353	296.3055
4	*S*(.) *f*(age-3)	302.5107	1.9744	0.09468	0.3726	4	110.3299	294.3002
5	*S*(.) *f*(age-5)	302.6819	2.1456	0.08691	0.342	6	106.2648	290.2351
6	*S*(.) *f*(age-4 w/1st 2 “age” classes = 2 days)	303.1178	2.5815	0.06989	0.2751	5	108.8301	292.8004
7	*S*(age-3) *f*(.)	303.7303	3.194	0.05145	0.2025	4	111.5496	295.5198
8	*S*(.) *f*(age-4)	303.8895	3.3532	0.04752	0.187	5	109.6018	293.572
9	*S*(age) *f*(age)	322.153	21.6167	0.00001	0	22	88.299	272.2692
10	*S*(age) *f*(.)	334.1492	33.6129	0	0	20	105.3514	289.3216
11	*S*(.) *f*(t)	338.823	38.2867	0	0	36	65.992	249.9623
12	*S*(.) *f*(age)	355.1442	54.6079	0	0	34	88.299	272.2692
13	*S*(*t*) *f*(.)	356.0698	55.5335	0	0	31	97.9278	281.898
14	*S*(*t*) *f*(*t*)	369.3462	68.8099	0	0	47	60.6821	244.6523

Given the relatively nearshore release locations (see Fig. 1) of the carcass assemblies, we had an a priori expectation that beaching probability might be higher within a relatively short (but unknown) period of time after release. As a result, in addition to fully age-dependent models (e.g., Model 9: S(age) *f*(age)), we also tested several models with a limited number of age-dependent beaching probabilities. One model, for example, assumed the daily probability of beaching was different on day 1, but the same on every day thereafter. This would be the case if the likelihood of carcass beaching was higher within the first 24 h, then be lower and relatively constant thereafter (e.g., Model 3 with *f*(age-2), i.e., two age-dependent beaching probability parameters). Another model assumed that beaching probability might be the same on the first 2 days, different on the third, and the same on all subsequent days (Model 2 with *f*(age-4 w/1st “age” = 2 days)). We expected daily survival probabilities to be less dependent on carcass age, so we tested only two models with age-dependent survival: one with full age dependence but constant beaching probability (i.e., Model #10), and one with three age categories (i.e., day 1, day 2, and thereafter; e.g., Model 7). We used Akaike’s information criterion for small sample sizes (AIC_c_) estimated using Program MARK (White and Burnham 1999) to select the best-supported model amongst the list of candidate models.

The Brownie band recovery model requires the following assumptions:

- The fates of individual carcasses were independent of one another, and
- The fate of a given carcass was a multinomial random variable.

Both of these assumptions were reasonable given our data, but we performed goodness-of-fit (GOF) tests on the data to check for deviance from expected model input (Burnham and Anderson 2002).

Data from the carcass drift study also required certain assumptions in order to apply the Brownie model in the context of the DWH oil spill and render the model results a valid estimate of the overall probability of beaching during the spill. The data assumptions included the following:

- Assumption 1:
- Barges did not affect the at-sea persistence or trajectory of carcasses
- Assumption 2:
- Transmitters did not fail or detach from carcasses except in cases of the carcass being eaten or other natural carcass loss processes
- Assumption 3:
- The date of first contact by ground crews was a reasonable approximation of the date of arrival of carcasses onshore
- Assumption 4:
- Ground crews found all carcasses that came ashore within the study area,
- Assumption 5:
- All telemetered carcasses found by ground crews and categorized as “beached” could have potentially been found by spill responders in an actual spill-response situation
- Assumption 6:
- Carcasses found in a foot-only state had been scavenged or otherwise decayed prior to their arrival onshore and
- Assumption 7:
- Carcass-release locations were representative of where birds died at sea during the DWH oil spill

These assumptions are supported by the data, but we nonetheless tested the degree to which violations could affect our final beaching probability estimates. We conducted sensitivity analyses to determine the importance of Assumptions 3 and 5 numerically. For Assumption 3, we tested the degree to which using earlier beach arrival times than when ground crews made first contact with carcasses would alter our overall beaching probability estimate. We measured model sensitivity to violations of Assumption 5 by recategorizing some of the carcasses found by our ground crews as permanently lost, under a more stringent definition of “findable by spill-response searchers.” Details of these analyses can be found in the online Supplemental Information, but we present a summary of results in “Results.” We also analyzed how sensitive our results would be to violations of Assumption 2 (transmitters did not fail or detach from carcasses), and Assumption 6 (carcasses found in a foot-only state had been scavenged or otherwise decayed prior to their arrival onshore). We discuss the results of these more qualitative assessments in “Discussion” along with justifications for the remaining assumptions.

## Results

Of the 14 candidate models considered, the four best models, with the lowest AIC_c_ values and all within 2 units of the top model, had a single daily at-sea survival probability (Table 1). This indicates strong support for a carcass’ chance of remaining afloat at sea being independent of the date or age of the carcass. While the best model also had a single daily beaching probability, the other three most-supported models included some form of age dependence in the beaching probabilities. For example, the second-best model provided separate beaching probability estimates for the first 4 days (with days 1 and 2 being the same), then a uniform beaching probability for all days thereafter. Our model selection analysis indicated no support for a model with full age dependency in the beaching probability, which would require 35 separate beaching probability parameters representing each possible day of beaching on day 1, day 2, …, up to day 35 (the duration of the carcass drift study). Time dependence was not supported in any of the top models for beaching or at-sea survival probabilities. Goodness-of-fit tests indicated that there was no strong evidence for lack of fit in our data relative to model assumptions (*P* = 0.182).

**Fig. 5:**
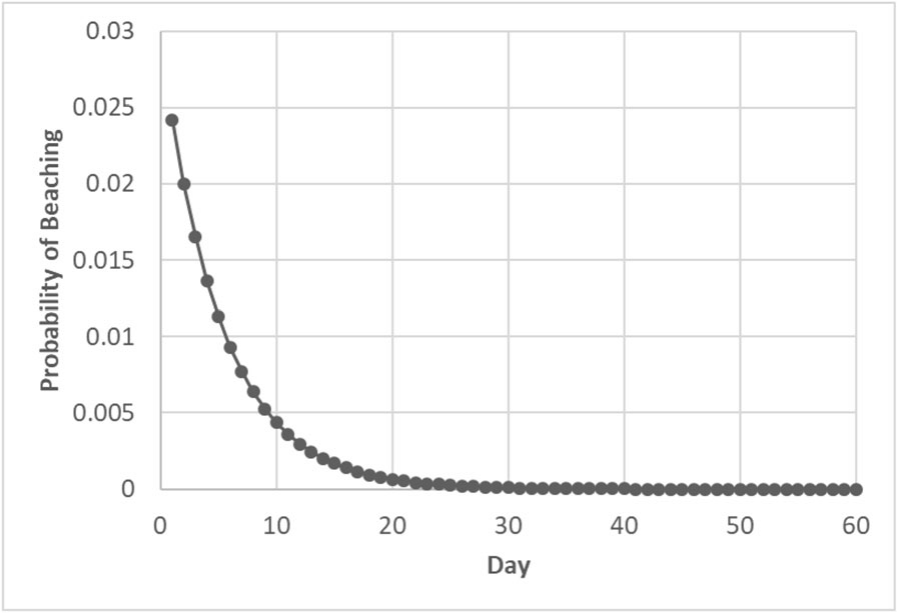
Graph illustrating the probabilities that a carcass would remain afloat at sea then beach on a given day (*b*_*i*_). The sum of the probabilities of each of these scenarios (0.1414) represents the overall probability of beaching (*β*)

The best-supported model in our candidate set of models estimated the daily probability of remaining afloat at sea (*S*) as 0.8290 (CI 0.75–0.89) and the daily probability of beaching (*f*) as 0.0242 (Cl 0.01–0.04). From these estimates, we calculated that the daily probability that a carcass would sink, be consumed, or otherwise become permanently lost (*ω*) was 0.1468 (i.e., 1–0.829–0.0242). Using the model-derived daily probability estimates, we calculated the daily probability of beaching after floating at sea for all preceding days (Fig. 5), then summed all possible daily probabilities and estimated that the overall probability of a carcass adrift at sea coming ashore at any point (*β*) was 0.1414.

### Quantitative sensitivity analysis results

When we reran all fourteen of our candidate models using altered datasets created under different assumptions regarding carcass arrival times (Assumption 3) and the findability of carcasses (Assumption 5), the most-supported models, as measured by AIC, were qualitatively the same as those supported under the original dataset. A single at-sea survival probability for all dates and ages of carcass was supported as was a single or partially age-dependent beaching probability (i.e. 1–4 beaching probability age categories). If our initial analysis violated Assumption 3 and carcasses came ashore earlier than the date of first contact by ground crews, estimates from our best model would yield an overall beaching probability of 0.139. If Assumption 5 were violated by our initial analysis and some carcasses (*n* = 5) were in fact inherently unfindable by spill-response type searchers because they were very heavily degraded or were located within areas not generally searched by spill-response personnel, then our overall beaching estimate would be 0.1147.

## Discussion

### Model assumptions

Our results indicate that a bird dying on the open water or on a tidally inundated substrate within the study area during the DWH spill would have had about a 14.14% chance of beaching. While the accuracy of this estimate depends on the degree to which the data adhere to the assumptions of our models, our sensitivity analyses (see Supplemental Information) indicated that the estimate of 14.14% was relatively insensitive to violations of Assumptions 3 (the date of first contact by ground crews was a reasonable approximation of the date of arrival of carcasses onshore) or 5 (all telemetered carcasses found by ground crews and categorized as “beached” could have potentially been found by spill responders). If those assumptions were violated, the chance of beaching would be in the range 11–13%, rather than 14%. Below, we briefly discuss the five remaining assumptions.

Assumption 1 states that barges containing transmitters did not affect the at-sea persistence of the telemetered carcasses. To the extent that carcasses were scavenged by invertebrates, we cannot imagine a mechanism by which the rate of carcass consumption could be affected by the barge assemblies. Vertebrates such as seabirds, alligators, or sharks might have been attracted or repelled by their novelty, but since scavengers in the area are obviously accustomed to floating debris, this also seems unlikely. Although the barge assemblies were constructed so that they did not have enough buoyancy to keep a carcass afloat, they clearly buoyed up small skeletal fragments, such as leg bones, that would otherwise have sunk. This may have caused heavily scavenged carcasses to float longer than they otherwise would have, resulting in an increased likelihood of beaching. Assumption 6 (carcasses found in a foot-only state had been scavenged or otherwise decayed *prior* to their arrival onshore and would not have beached without the added buoyancy of the transmitter assemblies) means that these fragments were not counted as beached carcasses.

Because carcasses were intensively searched for and our use of telemetry provided a significant advantage relative to bird recovery teams operating during the spill, we can reasonably assume that ground crews eventually located all carcasses that were beached where they could have been found during the spill response. It is therefore unlikely that Assumption 4 (ground crews found all carcasses that came ashore within the study area) was violated. This assumption is important because unfound carcasses would have been categorized as permanently disappeared when they had actually beached, resulting in an estimate of the at-sea loss rate that was biased low.

As described in “Methods,” our study design took into consideration the likely location of seabird deaths based on the density of live seabirds within the Gulf during the oil spill. Since our carcass release points reflected these locations, Assumption 7 (carcass release locations were representative of where birds died at sea during the DWH oil spill) is also unlikely to be violated in any substantial way.

While we have no evidence of any equipment failure, violation of Assumption 2 could potentially bias our results. The impact of such a violation was difficult to assess using an empirical approach because failures were not observed in this study or in the Alaskan study (Ford et al. 1996). We therefore used rough estimates of potential failure rates in order to test model sensitivity (see Supplemental Information) to this assumption. We found that transmitter failure rates of 5–10% would result in an estimated overall beaching probability of 15–16% rather than 14%.

For the 24 carcasses found beached in a “foot-only” state that were categorized as permanently lost, it is conceivable that some were actually beached as full or partial carcasses and were reduced to a foot-only state after beaching. Violation of Assumption 6 would cause our beaching probability estimate to be biased low. However, using additional study data for onshore scavenging rates (see Supplemental Information), we determined that it was unlikely that the 24 foot-only carcasses arrived onshore in a more intact condition. Our analysis suggested that at most, only one of those foot-only carcasses might have been scavenged onshore. If we incorrectly categorized a single carcass as permanently lost when it should have been categorized as beached, our estimated beaching probability would increase by less than 1% to something under 15%.

In general, analyses of our model assumptions indicate that the beaching probability estimate of 0.1414 is insensitive to violations of our assumptions, potentially overestimating the true beaching rate by a maximum of 0.03, or underestimating by a maximum of 0.02 if some of our model assumptions are violated. If all assumptions have an equal likelihood of being violated, our estimate has an equal likelihood of going up or down and by about the same magnitude in either direction. Any bias introduced by violations of our assumptions results in an estimate well within the confidence limits of our original estimate.

Although estimates from the present study are based on conditions in the Gulf of Mexico during July and August of 2011 when our field study was conducted, conditions in 2011 were similar to those in 2010 when the DWH spill occurred, and both 2010 and 2011 were typical years for the region based on wind speeds, temperatures, and sea level pressure (see undergroundweather.com for inter-annual comparative data). Other than these basic measures, we know of no environmental metric that could readily be used to compare years in terms of the advection and decomposition of dead birds between years, and extent to which results could vary between apparently similar years in the Gulf of Mexico remains an open question. We did, however, make every attempt in our 2011 field study and subsequent analysis to simulate conditions that were present during the 2010 spill and spill-response (e.g., initial carcass locations and location and extent of study area.) in order to render our results applicable to the DWH spill situation. A corollary to this is that our estimated beaching probability should not be construed as a universal estimate of the probability of beaching within the Gulf of Mexico, but only as an estimate of the overall beaching probability within the DWH spill-response area at the time of the spill response.

### Comparison with other drift studies

Estimates of the rate of at-sea carcass loss were first used as a part of avian injury quantification in the 1986 Apex Houston spill in California. This analysis utilized a trajectory model to determine where birds would have encountered oil and how long they would have floated before beaching. At the time, there were very few data available regarding how long a carcass persists at sea, consisting of a small amount of information from the *Hamilton Trader* spill in the Irish Sea (Hope Jones et al. 1970). These data suggested that the probability that a carcass would remain afloat each day was on the order of 0.85, and that this rate was constant no matter how long a carcass had been afloat. Subsequent experiments were carried out in the context of the Gulf of Alaska (Ford et al. 1996). In these experiments, some telemetered carcasses were released offshore of Kodiak Island where they were directed away from land by the local winds and currents, and some carcasses were released inside Prince William Sound where they were likely to strand on shorelines. Groups of carcasses released offshore typically floated with no attrition (i.e., an at-sea loss rate, *ω*, near 0) until, several weeks after their release, when they began to disappear at a rapidly accelerating rate until all were lost-at sea. This pattern of carcass disappearance was attributed to progressive water-logging of the carcasses which appeared to proceed at similar rates for all carcasses. Carcasses that were deployed within Prince William Sound behaved similarly to those deployed offshore. Many of these carcasses were stranded on shorelines, but most of these refloated on subsequent tides. Tracking crews who made contact with the stranded carcasses or with carcasses that were still afloat did not observe evidence of scavenging.

Although carcasses in the Alaskan study floated for weeks and then disappeared rapidly, carcasses in the Gulf of Mexico began to disappear the day that they were set adrift and continued to do so at a roughly constant rate (i.e., the best models supported a constant daily at-sea survival, *S*, of 0.829). Thus, in Alaska, little or no at-sea loss would occur if carcasses are beached within about 2 weeks of their death (Helm et al. 2015). In warmer subtropical waters like the Gulf of Mexico, however, at-sea loss appears to be more closely related to the length of time that carcasses floated before beaching. In Alaska, one would expect little or no at-sea loss within the first 5 days of being afloat, but by comparison, in the Gulf of Mexico, (using our estimate of the rate of loss at sea, *ω*, of 0.1468 per day), a carcass floating for 1 day would have a probability of 0.1468 of being lost at-sea, and would have had a 0.5224 probability of sinking over the course of 5 days (${\sum}_{x=1}^5{S}^{x-1}\omega$).

Differences between the Gulf of Mexico and the Gulf of Alaska regarding patterns of at-sea carcass loss probably reflect very basic differences in the oceanic systems themselves. The warmer waters of the gulf would both facilitate the rapid decomposition of the carcasses and encourage the populations of invertebrate scavengers that feed on them. We hypothesize that the primary cause of carcass loss in the Gulf of Mexico was scavenging, primarily by invertebrates, whereas in the Gulf of Alaska carcass loss resulted primarily from waterlogging. This interpretation is supported by the intact condition of carcasses found by ground crews in Alaska compared with the typically “bones only” or partially scavenged condition of carcasses recovered in the present study. If loss at-sea models based on either data collected in the Gulf of Alaska or in the Gulf of Mexico are to be used for NRDA purposes, care should be taken to select the correct model. In general, a high proportion of carcasses being stripped to the bones would suggest that the best model would be based on data from the Gulf of Mexico.

## Conclusions

In summary, our quantitative and qualitative sensitivity analyses suggest little evidence for large biases in our estimate of the daily at-sea loss rate (0.1468) or our estimate of overall beaching probability (0.1414) resulting from violations of our assumptions. The biases that could result if some of our assumptions were violated are likely to be counter balancing or in the direction of overestimation of the beaching probability. We therefore conclude that our primary estimate of 0.1414 for the overall beaching probability is a reasonable and well justified point-estimate, and we suggest that the 0.11 and 0.16 values derived from our exploration of potential model assumption violations provide good bracket estimates for *the probability that a seabird killed at sea during the DWH oil spill was beached in an area that was searched and in a state that was findable and identifiable by spill response personnel*. These estimates suggest that those carcasses that came ashore during the DWH spill represented only 11–16% of total birds killed by the spill.

The differences in the fate of avian carcasses in the Gulf of Alaska compared with the Gulf of Mexico are striking. Carcasses floating in the Gulf of Alaska—or likely other relatively cold waters—appear to become water-logged and sink, a process requiring about one to 3 weeks. The rate of carcass loss starts at zero during the first few days at sea and only slowly increases with time. In these circumstances, correction for loss at-sea due to sinking or scavenging may not be necessary if carcasses reach shore within a week or less. However, regardless of carcasses’ propensity to remain afloat, the pattern of ocean currents in the area of a spill may greatly increase at-sea loss if wind or currents tend to push carcasses offshore rather than toward shore. Based on our analysis, carcasses in the Gulf of Mexico—or likely other relatively warm waters—begin to disappear immediately and continue to disappear with a roughly constant probability of 0.1468 per day. Thus, in warm waters, there would be only a probability of 0.269 that a carcass would remain floating for a week (i.e., surviving at sea for 7 days, *S*^7^ = 0.829^7^). Corrections for at-sea loss due to sinking and scavenging therefore may be of great importance in relatively warmer waters.

## Electronic supplementary material

ESM 1 (DOCX 25 kb)
