# Supplementary material for: Using a mark-recapture model to estimate beaching probability of seabirds killed in nearshore waters during the Deepwater Horizon oil spill
Source: Environ Monit Assess. 2020 Mar 17;191(Suppl 4):813. doi: 10.1007/s10661-019-7919-9 (PMC7078145; doi:10.1007/s10661-019-7919-9)
Supplement: Supplementary file 1 — (DOCX 25 kb) [file 10661_2019_7919_MOESM1_ESM.docx]

**Supplementary Information**

**Using a Mark-recapture Model to Estimate Beaching Probability of Seabirds Killed in Nearshore Waters during the Deepwater Horizon Oil Spill**

Gina K. Himes Boor, R. Glenn Ford

Gina K. Himes Boor

Ecology Department

Montana State University

Bozeman, Montana 59715

[gkhimesboor@montana.edu](mailto:gkhimesboor@montana.edu)

R. Glenn Ford

R.G. Ford Consulting Company

Portland, Oregon 97232

[eci@teleport.com](mailto:eci@teleport.com)

Model Assumptions

In order to carry out our analysis of beaching rates using the Brownie model, we needed to make several assumptions about our data. These assumptions are as follows:

1) barges did not affect the at-sea persistence or trajectory of carcasses,

2) no equipment failure occurred (i.e., no detachment of transmitters from carcasses occurred except in cases of the carcass being eaten or other natural carcass loss, and no transmitters failed during the course of the study),

3) the date of first contact by ground crews was a reasonable approximation of the date of arrival of carcasses onshore,

4) ground crews found all carcasses that came ashore within the study area,

5) all carcasses found by ground crews and categorized as “beached” had the potential to be found by searchers in an oil spill response situation (i.e., they were not inherently unfindable),

6) carcasses found in a feet-only state had been scavenged or otherwise decayed prior to their arrival onshore, and

7) the release locations used in the carcass drift study and for this analysis were representative of where birds died at sea during the Deepwater Horizon oil spill.

We conducted several post-hoc analyses to determine how violations of some of the assumptions would impact our final beaching probability estimate (e.g. a sensitivity analyses). Below we describe each of these post-hoc analyses in detail as they pertain to each of the assumptions, and to provide estimates of how much violations of our model assumptions would alter our final beaching probability estimate. Note, we did not conduct analyses for all of the assumptions (see Discussion section in main manuscript for information on assumptions not addressed below).

Assumption 2: Equipment Failure

We have no evidence to suggest from this or from previous studies that we had any equipment failure with our barges or attachments. Given the many barges attached only to carcass feet (n=24) that we found during the study, it appears that attachment failure was highly unlikely, even after legs were no longer attached to carcasses. Since we do not have an estimate of the likelihood of telemetry failure, it is difficult to estimate the exact impact of this type of equipment failure. However, to understand how sensitive our overall beaching probability estimate is to a departure from the assumption of no equipment failure, we conducted an analysis assuming two alternative levels of potential equipment failure (5% and 10% failure rate). If our barges failed at a 5% or 10%) rate, either from attachment or telemetry signal failure, , a total of 10 or 20 barges respectively would have failed. All of these would have been categorized as “permanently lost” carcasses in our primary analysis. If we simply exclude those from the analysis, our proportion of beached birds would be 27/185 = 0.14.6 or 27/175 = 0.154. Comparing those proportions to the same metric under our initial assumptions, 27/197 = 0.138, which represents a a rough estimate of the beaching probability and is a close approximation of our model-based estimate of 0.1414, we can determine the general magnitude of impact that a violation of assumption 2 would have on our model-based estimate. Using these metrics, violation of assumption 2 would make our beaching probability estimate an underestimate of the true beaching probability by approximately 0.01 or 0.02). This result indicates that even with a 10% failure rate (much higher than we believe occurred) of our barge units, our final beaching probability would be minimally impacted.

Assumption 3: Carcass ‘Arrival Time’

Under assumption 3, we assume that the date of first contact with a carcass by study ground crews is a reasonable approximation of when the carcass arrived onshore. The dates of arrival onshore affect the estimates for the probability of beaching and surviving at sea. In considering whether our data meet this assumption we must take into account the methodology and circumstances of the carcass drift study. During the study, active transmitters were regularly relocated by the tracking aircraft. But given the complexity of the shoreline in many parts of the study area, their estimated positions did not allow us to confidently determine whether transmitters were on land or still in the water. Therefore, it was necessary for ground crews to find the telemetered carcass onshore in order to be certain that the carcass had actually beached. The protocol during the study was for aerial crews to alert ground crews of the approximate location of a transmitter when they detected a telemetry signal in the vicinity of the shoreline. The aerial tracking crews generally could not see the carcass and barge assembly, but could provide an approximate location based on the telemetry signal. On some occasions, ground crews in search of potential beached carcasses could not yet detect them onshore, and in other instances carcasses were found onshore soon after aircraft crews noted that they were nearshore and were potentially beached. Occasionally, ground crews were unable to immediately travel to the location of a suspected beached carcass due to the distance from the crew to the transmitter or because of access restrictions in the suspected beaching area. But because of the lack of precision in the aircraft based locations, delays of the ground crews probably did not cause the recorded beaching date to be late since carcasses may still have been in the water and not yet beached at the time the ground searches were initiated.

If carcasses tended to arrive significantly earlier than the date of first ground contact, our daily at-sea survival and beaching estimates would be biased, resulting in a biased estimate of the beaching probability. To assess the direction of the potential bias and the degree to which the estimated probability of beaching might be affected, we reran our set of candidate models using the same carcasses retrieved by ground crews (n=27), but instead of using the date of *first* ground crew contact as their recovery dates, we used the date on which each carcass *might* have come ashore based on approximate location data from the tracking aircraft. Because the locations reported by aerial crews were not precise, we defined “reaching shore” as the first date on which a carcass’ approximate location (from aerial tracking) was within 500 m of a shoreline as determined using Google Earth maps.

The best supported models based on AICc scores suggested a single at-sea “survival” value for all carcasses no matter the date on which they were released or how long they had been afloat at sea. The best models suggested that beaching probability remained constant over time and age of carcass, or that the beaching probability differed among carcasses of age 1, 2, or 3 days only. Overall beaching probability estimates derived from the at-sea survival and beaching probability estimates from these top models did not differ substantially from one another, and was estimated at about 0.1385. This suggests that, if carcasses consistently came ashore earlier than the first ground contact, the overall beaching probability estimate derived from our primary model (0.1414) would be an overestimate of the true value. Because of the uncertainty associated with aerial-based determination of the date of arrival onshore, the assumption that carcasses beached on the day on which they were first encountered by ground crews, is the more readily justifiable. Nonetheless, some carcasses may have come ashore earlier than the first contact, and therefore the true overall beaching probability could be slightly lower than our estimated value.

Assumptions 4 & 5: Potential Findability

Because drift study carcasses were located using telemetry signals, we can assume that ground crews were able to locate all the carcasses that beached within the study area (assumption #4). This assumption is important because if ground searchers did not find all carcasses in the study area, those carcasses would have been categorized as permanently disappeared when they had actually beached. Given our use of telemetry and the intense search effort and tracking by aircraft, boat, and ground, we have no reason to believe our data demonstrate any departure from this assumption. The thoroughness of the searches made possible by telemetry, however, may have contributed to a violation of the assumption that all carcasses found onshore during the drift study were *potentially* findable in an oil spill response situation (assumption #5). In some cases, telemetered carcasses were located even when they were under water, in deep emergent vegetation, buried in sand or mud, or nearly impossible to find even with telemetry (e.g., ground crews returned multiple times before finding the carcass). We therefore defined “potentially findable” as being in a state and location where spill-response searchers might find it. During the spill response, searchers did not dig for carcasses or search under water. Drift study carcasses that were buried under deep sand or mud or under water were therefore *not* categorized as potentially findable and were counted as “permanently lost”. Similarly, transmitters that were detected but could not be located or accessed by ground crews due to restrictions were not considered potentially findable, and were categorized as “permanently lost”). Drift-study carcasses that were found in deep emergent vegetation and/or were only slightly more than “feet-only” were counted among the 27 beached carcasses. Because these carcasses were found within the study area and met our definition of a “recognizable carcass” (i.e., more than just feet or leg bones), we included them with other carcasses that were categorized as having beached. But these carcasses may not actually have been “potentially findable” by oil spill response searchers. If these carcasses were *not* potentially findable, then our overall beaching probability estimate will be positively biased, and the estimated likelihood of beaching will be too high.

To understand the degree to which our estimate could be positively biased by a lack of findability, we conducted an assessment of the likelihood that each of the 27 “beached” carcasses could have been found by spill-response searchers based on the state of each carcass (e.g., amount of carcass present, state of deterioration), its location (e.g., geographic location, habitat type), and the level of entanglement in vegetation or other debris. Based on this assessment, we estimated that between 42% and 75% (n=10 to 18) would not have been found by spill-response searchers. While we think these estimates are realistic, they have a significant element of subjectivity. To better quantify the extent to which our overall probability of beaching might be biased by findability issues, we used an objective but very conservative definition of “unfindable” to create a new dataset of “beached” carcasses and reran our set of candidate models. The new dataset had 5 fewer carcasses in the “beached” category compared to our original data (for a total of 22 beached carcasses). The 5 carcasses that we defined as “unfindable” by spill response searchers included 3 carcasses with minimal remains (but slightly more than feet-only), and 2 carcasses found in rip-rap, which was rarely searched during the spill response.

The best supported models again suggested single at-sea survival and beaching parameters, or 2 to 4 age-dependent beaching parameters. The overall beaching probability estimate derived from the at-sea survival and beaching probability estimates from the best supported model using this modified dataset was 0.1147. This result suggests that our original estimate of 0.14 may overestimate the overall beaching probability by 0.027 if our assumption that all carcasses found onshore during the drift study were “potentially findable” was violated by these 5 carcasses. If our assumption is violated by any of the other carcasses we deemed unfindable, then our original estimate will be overestimated by even more.

Assumption 6: Potential Onshore Scavenging

The fact that the ground crews may not have always retrieved a carcass within a day of its beaching affected other aspects of the analysis as well. For modeling purposes, we assumed that transmitters found attached only to legs bones or feet arrived onshore in that condition (assumptions 6). However, carcasses that were *not* located immediately could potentially have been reduced to a feet-only state *after* coming ashore. If this were the case, our overall beaching probability estimate of 0.1414 would be an underestimate. To assess whether the assumption is warranted, we used post-beaching data from 18 carcasses that were located by ground crews but left in place and periodically checked for disappearance or deterioration. We used this post-beaching information to assess the frequency with which carcasses degraded to a feet-only state while on shore and the speed at which the degradation occurred. Of the 18 carcasses left in place after first ground contact, only 3 (16.7%) were ever reduced to a feet-only state on shore. It took 3, 5, and 13 days for each of the three carcasses to be reduced to a feet-only state while onshore.

To apply these carcass persistence data to the 24 unburied feet-only carcasses in our dataset, we assessed possible inaccuracies in the time of beaching of the 24 feet-only carcasses. Using the approximate locations provided by the aircraft crews, we assumed that any estimated transmitter location within 500 m of shore was actually onshore. Using this criterion for beaching, we found that only 8.3% (n=2) could have been onshore for more than 3 days before being located by ground crews. Another 20.8% (n=5) of the feet-only carcasses could have been onshore for 3 days. If these 2-7 feet-only carcasses each had a 0.167 probability of being reduced to feet-only while onshore, we would expect that 1 or fewer would have been scavenged onshore and reduced to a feet-only state. Based on this assessment we conclude that our assumption that feet-only carcasses arrived onshore in feet-only condition and therefore were permanently lost is well justified and any departures from this assumption are likely to be minimal (i.e., would likely add 1 or no carcasses to the “beached” category).

We used a rough estimate of beaching probability based on the proportion of beached carcasses out of total carcasses in the study (27/195 = 0.138 under our original assumptions, and compared that with the same beaching probability metric assuming that one of the feet-only carcasses classified as permanently lost was classified as beached (28/195 = 0.144). In the unlikely event that assumption 6 is violated, our model-derived estimate would increase by about the same magnitude as the difference between these two rough metrics. In such a case, we would expect our overall beaching probability estimate to be underestimated by about 0.006.
